# Neural correlates of visual and tactile path integration and their task related modulation

**DOI:** 10.1038/s41598-023-36797-8

**Published:** 2023-06-19

**Authors:** Lisa Rosenblum, Alexander Kreß, B. Ezgi Arikan, Benjamin Straube, Frank Bremmer

**Affiliations:** 1 Department Neurophysics, Philipps-Universität Marburg, Karl-Von-Frisch-Straße 8a, 35043 Marburg, Germany; 2 Center for Mind, Brain and Behavior, Philipps-Universität Marburg and Justus-Liebig-Universität Giessen, Giessen, Germany; 3 Translational Neuroimaging Marburg, Department of Psychiatry and Psychotherapy, Philipps-Universität Marburg, Marburg, Germany; 4 Department of Psychology, Justus-Liebig-Universität Giessen, Giessen, Germany

**Keywords:** Perception, Sensory processing

## Abstract

Self-motion induces sensory signals that allow to determine travel distance (path integration). For veridical path integration, one must distinguish self-generated from externally induced sensory signals. Predictive coding has been suggested to attenuate self-induced sensory responses, while task relevance can reverse the attenuating effect of prediction. But how is self-motion processing affected by prediction and task demands, and do effects generalize across senses? In this fMRI study, we investigated visual and tactile self-motion processing and its modulation by task demands. Visual stimuli simulated forward self-motion across a ground plane. Tactile self-motion stimuli were delivered by airflow across the subjects’ forehead. In one task, subjects replicated a previously observed distance (Reproduction/Active; high behavioral demand) of passive self-displacement (Reproduction/Passive). In a second task, subjects travelled a self-chosen distance (Self/Active; low behavioral demand) which was recorded and played back to them (Self/Passive). For both tasks and sensory modalities, Active as compared to Passive trials showed enhancement in early visual areas and suppression in higher order areas of the inferior parietal lobule (IPL). Contrasting high and low demanding active trials yielded supramodal enhancement in the anterior insula. Suppression in the IPL suggests this area to be a comparator of sensory self-motion signals and predictions thereof.

## Introduction

Self-motion through an environment induces various sensory signals that allow for the estimation of parameters such as traveled distance, direction (heading), and speed. The visual pattern induced by self-motion is called optic flow^1,2^. Path integration describes the ability to estimate the traveled distance (and angular direction) of one’s self-motion with respect to a reference point. The integration of optic flow over time allows the moving observer to determine the distance that has been traveled^3,4^. For the tactile modality, a previous behavioral study has shown that the estimation of traveled distance is feasible by the integration of tactile flow over time in a distance replication task^5^.

Neural correlates of path-integration have been mainly studied in the visual modality. Key cortical regions for visual self-motion processing are areas hMST^6,7^, hVIP^8,9^, hCSv^10^ and hV6^11^. But also auditory self-motion cues (pitch scaling with speed) can be used to reproduce traveled distance^12^. Based on this result, in a follow-up fMRI study employing visual and auditory self-motion stimuli, we have shown that the active reproduction compared to the passive encoding of a travel distance leads to enhanced BOLD activity in early sensory (visual and auditory) cortices. In addition, we found suppressed BOLD response in higher-order areas such as the angular gyrus^13^. The finding of enhanced BOLD response in early cortical areas was somewhat surprising given that the predictive coding framework hypothesizes the attenuation of neural responses for self-generated (i.e., predicted) signals (but see also Refs.^14,15^ for evidence of response enhancement). We suggested that enhanced BOLD response in early sensory cortices might have been driven by engagement of attentional and working memory resources during the on-line comparison of traveled and target distance to solve the task of reproduction. In this sense, attention operates as a top-down mechanism that increases the precision of perceptual inference^16–18^ and optimizes the correctness of prediction errors by reversing the effect of prediction^19–21^. We proposed that in that context, behavioral task demands led to an enhanced BOLD response in early cortices while BOLD activation was suppressed in higher-level areas reflecting conformity of predictions and information about traveled distance^22,23^. However, in our previous study, behavioral task demand was not manipulated as an experimental factor.

In the present study, we investigated the neural correlates of tactile and visual self-motion processing in a path-integration task using human fMRI. We aimed to further examine how the perception of traveled distance is influenced by task-demands by manipulating it as an experimental factor. Subjects solved a path integration task using either visual or tactile flow. High behavioral demand was induced by a Reproduction (Repro) task, where subjects actively replicated (Active; *high* monitoring demand) a previously observed self-displacement (Passive; *high* encoding demand). As a low demanding task, we introduced the Self task (Self), where subjects first traveled a self-chosen distance (Active; *low* monitoring demand) which was recorded and then played back to them, without a further task (Passive; *low* encoding demand). In Active trials of the Repro task (= Repro/*Act*), behavioral demand was expected to be higher compared to Active trials of the Self task (= Self/*Act*) given that subjects had to maintain a target distance in register and evaluate and compare traveled distance online to be able to successfully solve the task. Following our previous study on visual and auditory path-integration^13^, we expected to find enhanced BOLD response in early sensory areas and suppressed BOLD response in higher-order areas for Active as compared to Passive trials. Likewise, given the higher behavioral demand, we expected to find stronger BOLD activation in Repro/Act trials as compared to Self/*Act* trials.

## Results

### Behavioral results

**Figure 1:**
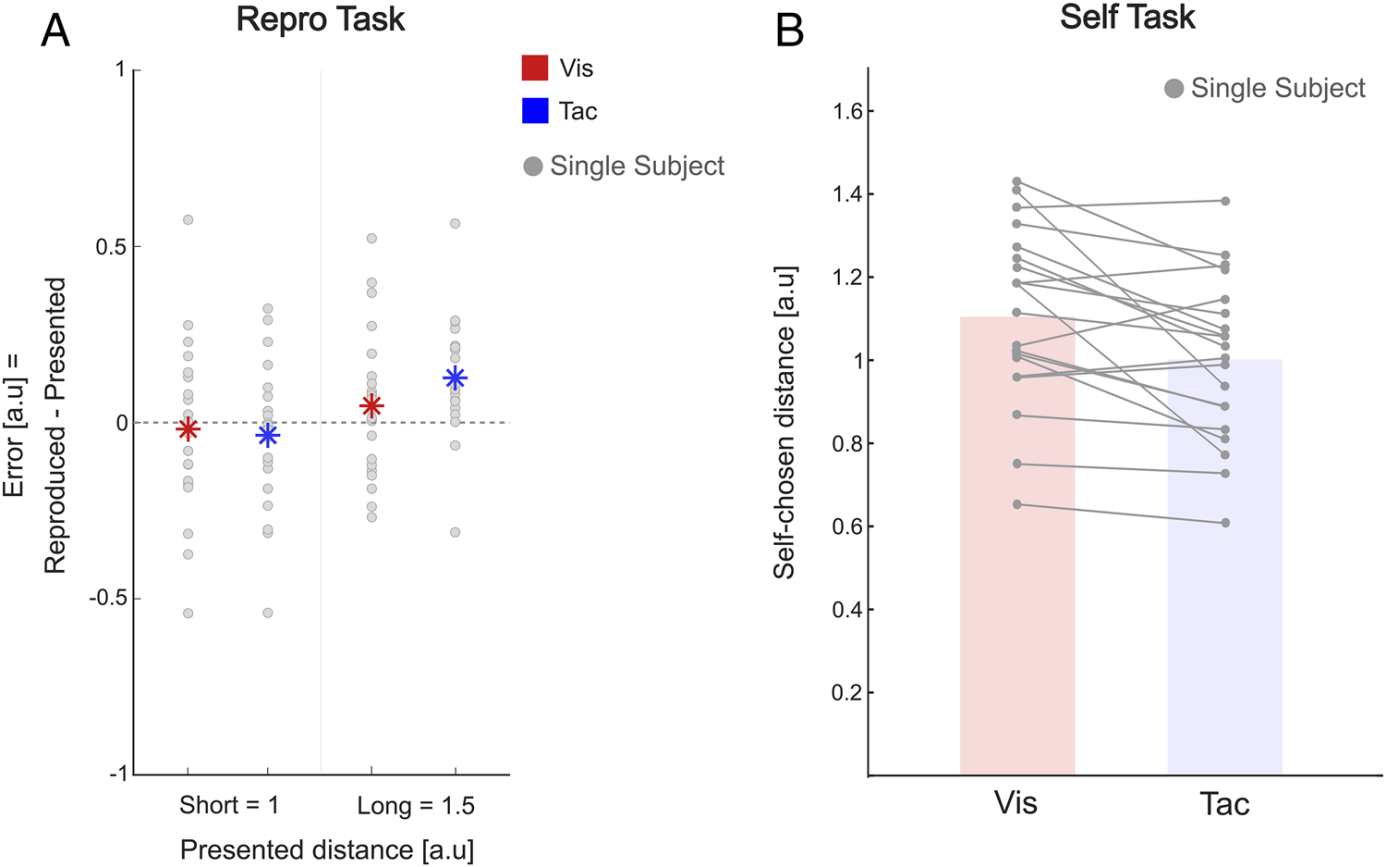
(**A**) Accuracy of reproduced distances in the Repro task. Mean Error, defined as reproduced distances (Repro/Act) minus presented distance (Repro/Pas), as a function of target distance (short and long). Asterisks represent mean across all subjects for the visual (red) and tactile (blue) conditions and single grey dots represent single subject data. Positive values indicate that subjects overshot the target distance, and vice versa. (**B**) Mean traveled distances in the Self task. Mean self-chosen traveled distances are shown on the y-axis for the visual (red bar) and tactile (blue bar) modalities. Like in A, dots represent single subject data, and lines connect data points from the same subjects.

For the Repro task where subjects actively reproduced a previously observed travel distance, we quantified the accuracy of replicated distance by computing the resulting ‘Error’ (Reproduced distance—Presented distance). Subjects were presented with two different target distances and always traveled at a constant speed. Here, the short distance of 1 in arbitrary units (a.u.) corresponds to a travel duration of 1 s and a long distance of 1.5 a.u. to a travel duration of 1.5 s. Errors were investigated for both presented distances in both modalities (Vis & Tac) separately. Figure 1A (left panel) shows the Errors (ordinate) for both target distances and both modalities (gray dots depict single subject data, while colored asterisks depict the mean). Positive values on the y-axis correspond to an overestimation of travel distances (overshoot) and negative values to an underestimation of travel distances (undershoot). While subjects’ responses revealed overall a slight undershoot for short distances (*Mean* Vis_Short_: − 0.02 a.u.s, *Mean* Tac_Short_: − 0.03 a.u.s.), long distances were overestimated by most subjects (*Mean* Vis_Long_: 0.06 a.u.s; *Mean* Tac_Long_: 0.1 a.u.s) [RM ANOVA, *F*(1, 20) = 16.558, *p* < 0.001, η^2^ = 0.453, main effect “Distance”]. Accuracy did not differ significantly between both modalities [RM ANOVA, *F*(1, 20) = 0.835, *p* = 0.372, η^2^ = 0.04, main effect “Modality”] indicating comparable performance between conditions. After the experiment, subjects were asked to indicate their reproduction strategy in an open response format. Counting and memorizing a rhythm as reproduction strategies was reported by most of the subjects.

In the Self task, subjects traveled self-chosen distances that were recorded and played back to them. Figure 1B shows mean traveled distances [a.u.] for the Vis and Tac trials separately (ordinate). Bars show the subject mean of the self-produced distances and dots depicting single subject data. The overall produced distances were slightly but significantly longer in the visual (*Mean* = 1.1 a.u.s, *SD* = 0.2 a.u.s) as compared to the tactile modality (*Mean* = 1.0 a.u.s, *SD* = 0.19 a.u.s) (paired samples t-test: *t*(20) = 3.194, *p* < 0.01). Traveled distances correlated between the visual and tactile modality across subjects, i.e., participants who traveled shorter (longer) distances in the visual modality also tended to travel shorter (longer) distances in the tactile modality (Pearson's corr. 0.734, *p* < 0.001).

### Imaging results

#### Neural correlates of path-integration

**Figure 2:**
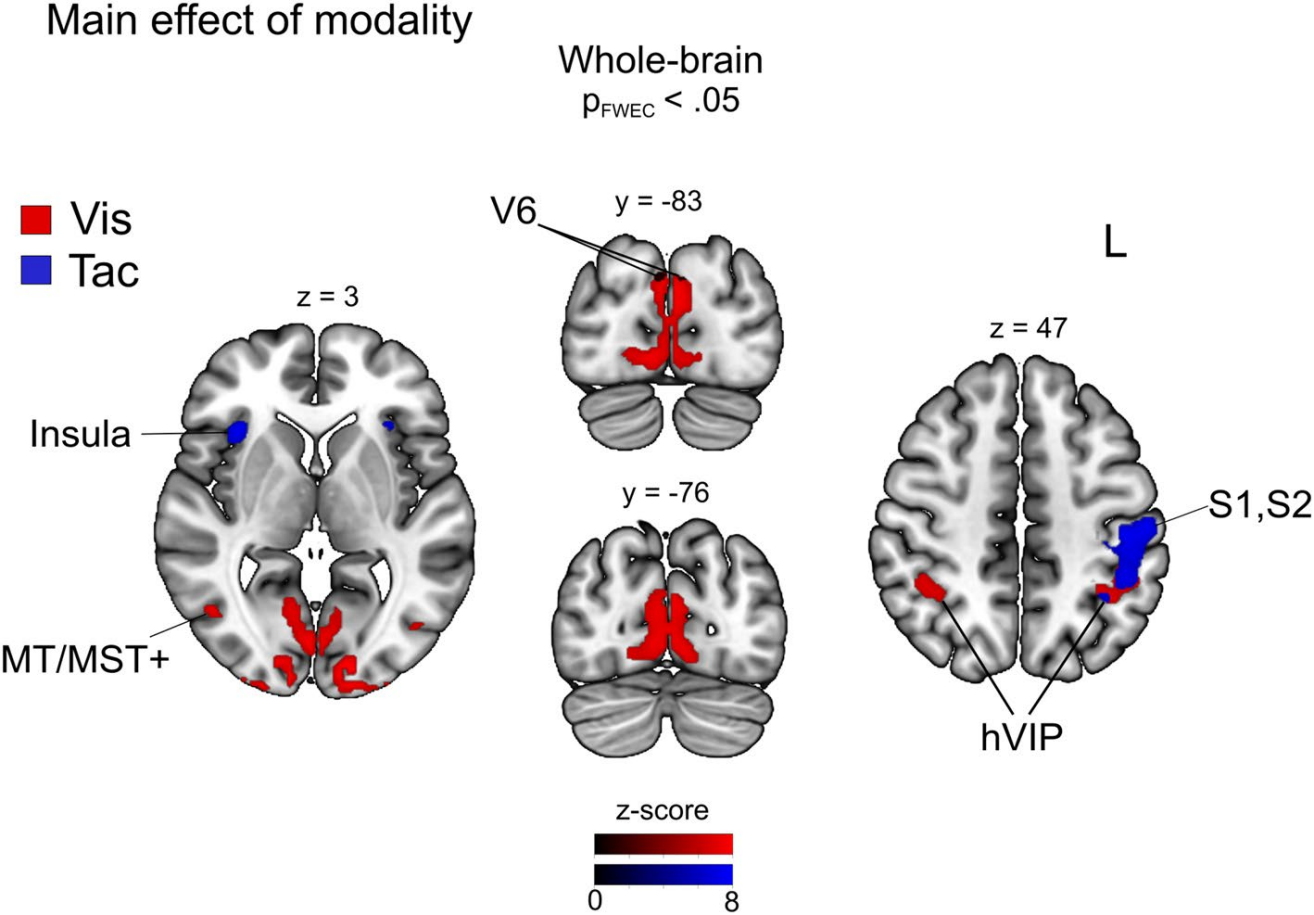
Neural responses induced by visual (red) and tactile (blue) self-motion stimuli. Clusters showing a significant main effect for visual and tactile self-motion encoding, respectively, across all tasks. Whole-brain results show BOLD responses in early and higher-order visual areas for visual optic flow stimuli and responses in early and higher-order somatosensory areas for tactile flow simulating forward self-motion, significant at *p*_*FWE*_ < 0.05.

To investigate the overall effect of sensory modality on BOLD activation during distance encoding, we conducted F-tests over visual and tactile trials irrespective of task condition (main effect against baseline). Given the nature of our self-motion stimuli, we expected to find a significant BOLD response in early visual cortex and in areas sensitive for visual self-motion stimuli as well as in early and higher somatosensory cortices for tactile stimuli, respectively. In particular, we expected to find higher BOLD response in the ventral intraparietal area (hVIP) for both stimulus modalities given the multimodal response properties of area hVIP [e.g., Ref.^8,24^]. Figure 2 displays the clusters for main effects of each modality (Vis = red, Tac = blue) and Table 1 reports the activation peaks in significant clusters. For visual trials, we observed significant activation in bilateral striate (V1) and extrastriate visual areas (V2, V3, MT-complex) and bilateral area hVIP^8^. Visual areas that have been found to encode different aspects of self-motion were investigated based on peak coordinates derived from previous studies. Specifically, we probed area V6^25^, cingulate sulcus visual area (CSv)^26^, and the hMST region^27^. For effects of interest depicted in Fig. 2, we have identified significant activation bilaterally in areas V6 and hMST on the peak-level only. For tactile trials, we found significant activations in the left primary and secondary somatosensory cortices (S1, S2), bilateral anterior insular cortex (AIC), and left area hVIP. At the uncorr. level (not shown in Fig. 2), we also found significant activation for right area hVIP, ([*x, y, z* = 34, − 56, 46], *k*_*E*_ = 140, *p*_*uncorr*_*.* < 0.01) and for right area S1 ([*x, y, z* = 46, − 32, 46], *k*_*E*_ = 86, *p*_*uncorr*_*.* < 0.01), but not S2.

**Table 1:** Anatomical locations of cluster activations for main effects of the visual and tactile modality, respectively. Coordinates are listed in MNI space. *p*_*FWE*_ < 0.05.

	Anatomical label	Cluster extent (anatomy toolbox)	Coordinates (peak of sign, cluster, MNI)					
			Side	x	y	z	*z* value	*k*_E_
*Vis*	Occipital pole	V1, V2, V3	R	30	− 94	− 6	> 8	1802
		V1, V2, V3	L	− 32	− 94	− 8	> 8	409
	Lateral occipital cortex	V5	R	44	− 62	2	6.32	22
			L	− 42	− 70	2	6.11	13
	IPS	hVIP	R	40	− 44	46	5.41	85
			L	− 34	− 50	46	6.21	214
*Tac*	Parietal lobe	Postcentral gyrus	L	− 44	− 18	58	7.18	420
	Parietal lobe	Postcentral gyrus	L	− 46	− 42	48	5.47	5
	Parietal operculum	hVIP, SII	L	− 44	− 42	48	5.74	117
	Insular cortex	Operculum	L	− 32	16	10	6.64	110
		Operculum	R	32	18	8	6.34	133
	Supramarginal gyrus	IPL	R	56	− 38	18	5.94	22
			L	− 42	− 34	20	5.38	15

### Modulatory effect of behavioral demand

**Figure 3:**
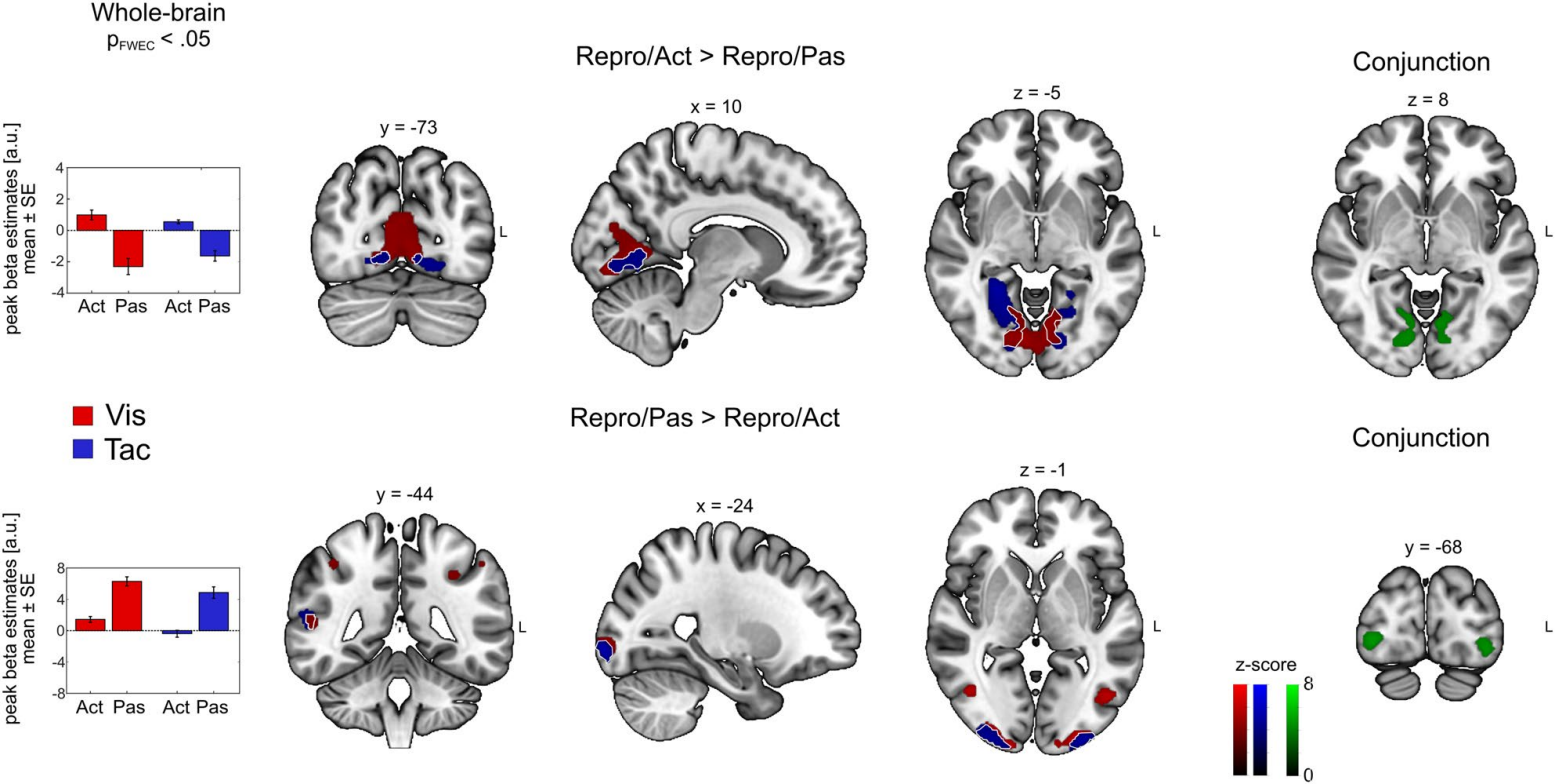
Modulatory effect of behavioral demand. Whole-brain results showing BOLD enhancement (top row) and BOLD suppression (bottom row) during the reproduction of target distances compared to passively encoding distances for visual (shown in red) and tactile (shown in blue) stimuli. Commonly enhanced or suppressed regions across visual and tactile stimuli are demarcated by a white line. Bar graphs show mean beta estimates across subjects (± *SE*) for the corresponding peak voxel. Clusters derived from a conjunction analysis across both modalities are shown on the right (green). Cluster-forming threshold for all maps was *p*_*FWE*_ < 0.05.

In the Repro task, contrasting Act against Pas trials (*Act* > *Pas*) for the visual modality yielded significant bilateral clusters in early visual sensory cortices. Surprisingly, for the tactile modality, this contrast also showed significant bilateral clusters in early visual sensory cortices. Figure 3 (top row) shows significant activation clusters for this contrast in each modality (Vis = red, Tac = blue) as well as corresponding mean beta estimates for the peak voxels. White lines demarcate overlapping activation in both conditions. A conjunction analysis over both modalities (Repro/*Act* > Repro/*Pas* visual ∩ Repro/*Act* > Repro/*Pas* tactile) showed significant bilateral activations in early visual cortices (Fig. 3, right column. Conjunction = Green). For unimodal visual trials and the conjunction analyses, we found large significant clusters that contained multiple anatomical regions. We reviewed the significant clusters by entering the corresponding contrast to the Anatomy Toolbox and by applying a V3A mask^28^ and found small, but significant activations of bilateral area V3A.

To identify possible BOLD suppression effects in Act relative to Pas trials, we calculated the contrast (*Pas* > *Act*) (Fig. 3, bottom row). For visual trials, we observed significant clusters in higher-order visual areas (e.g., V3, MT/V5, hVIP) and the inferior parietal lobe (IPL). For the tactile modality, the (*Pas* > *Act*) contrast showed significant bilateral clusters in higher-order visual areas located in the lateral occipital cortex (LOC) and the left IPL. A conjunction analysis over both modalities (Repro/*Pas* > Repro/*Act* visual ∩ Repro/*Pas* > Repro/*Act* tactile) showed significant bilateral clusters in V3 and the IPL. Table 2 reports all identified cluster peaks of the contrasts derived from the Repro task.

**Table 2:** Anatomical locations of cluster activations for contrasts of interest in the Repro task. Coordinates are listed in MNI space. Initial search threshold was *p* < 0.001, only regions passing the *p*_FWE_ < 0.05 at the cluster level are shown.

	Anatomical label	Cluster extent (anatomy toolbox)	Coordinates (peak of sign, cluster, MNI)							
			Side	x	y	z	*k*_E_	*z* value	p_FWE_ voxel	p_FWE_ cluster
*Repro/Act* > *Repro/Pas*										
* Vis*	Lingual	V1, V2, V3A	R	2	− 78	2	5882	6.38	< 0.001	< 0.001
* Tac*	Lingual	V1, V2, V3	R	26	− 48	− 4	7209	6.85	< 0.001	< 0.001
* Conj*	Lingual	V1, V2, V3A	R	12	− 62	0	3975	5.49	= 0.001	< 0.001
*Repro/Pas* > *Repro/Act*										
* Vis*	Occipital Mid	hOc4lp	L	− 30	− 96	− 6	2156	> 8	< 0.001	< 0.001
	Occipital Inf	hOc3v	R	30	− 94	− 6	702	7.57	< 0.001	< 0.001
	Temporal Mid	MT/V5	R	44	− 62	6	3336	5.91	< 0.001	< 0.001
	Precentral	Area 6d3	L	− 28	− 6	54	2191	5.32	< 0.010	< 0.001
	Parietal Inf	IPS	L	− 32	− 42	40	2586	5.17	< 0.010	< 0.001
	Frontal Inf Oper	Pars Opercularis	R	54	10	18	749	4.39	= 0.112	< 0.001
* Tac*	Occipital Mid	hOc4lp	L	− 30	− 96	− 6	1723	> 8	< 0.001	< 0.001
	Occipital Inf	hOc3v	R	30	− 94	− 6	408	7.27	< 0.001	< 0.010
	Temporal Sup	IPL	R	54	− 42	14	2323	5.43	= 0.001	< 0.001
	Angular	IPS	L		− 36	− 66	40	447	4.42	< 0.010
	Temporal Sup	IPL	L	− 56	− 38	22	247	4.23	= 0.201	< 0.050
	Precuneus	SPL	L	− 4	− 62	62	430	3.94	= 0.469	< 0.010
* Conj*	Occipital Mid	hOc4lp	L	− 30	− 96	− 6	1258	> 8	< 0.001	< 0.001
	Occipital Inf	hOc3v	R	30	− 94	− 6	388	7.27	< 0.001	< 0.010
	Temporal Sup	IPL	R	52	− 42	12	1160	5.33	< 0.010	< 0.001
	Parietal Inf	IPS	L	− 30	− 48	44	331	4.15	= 0.255	< 0.050

**Figure 4:**
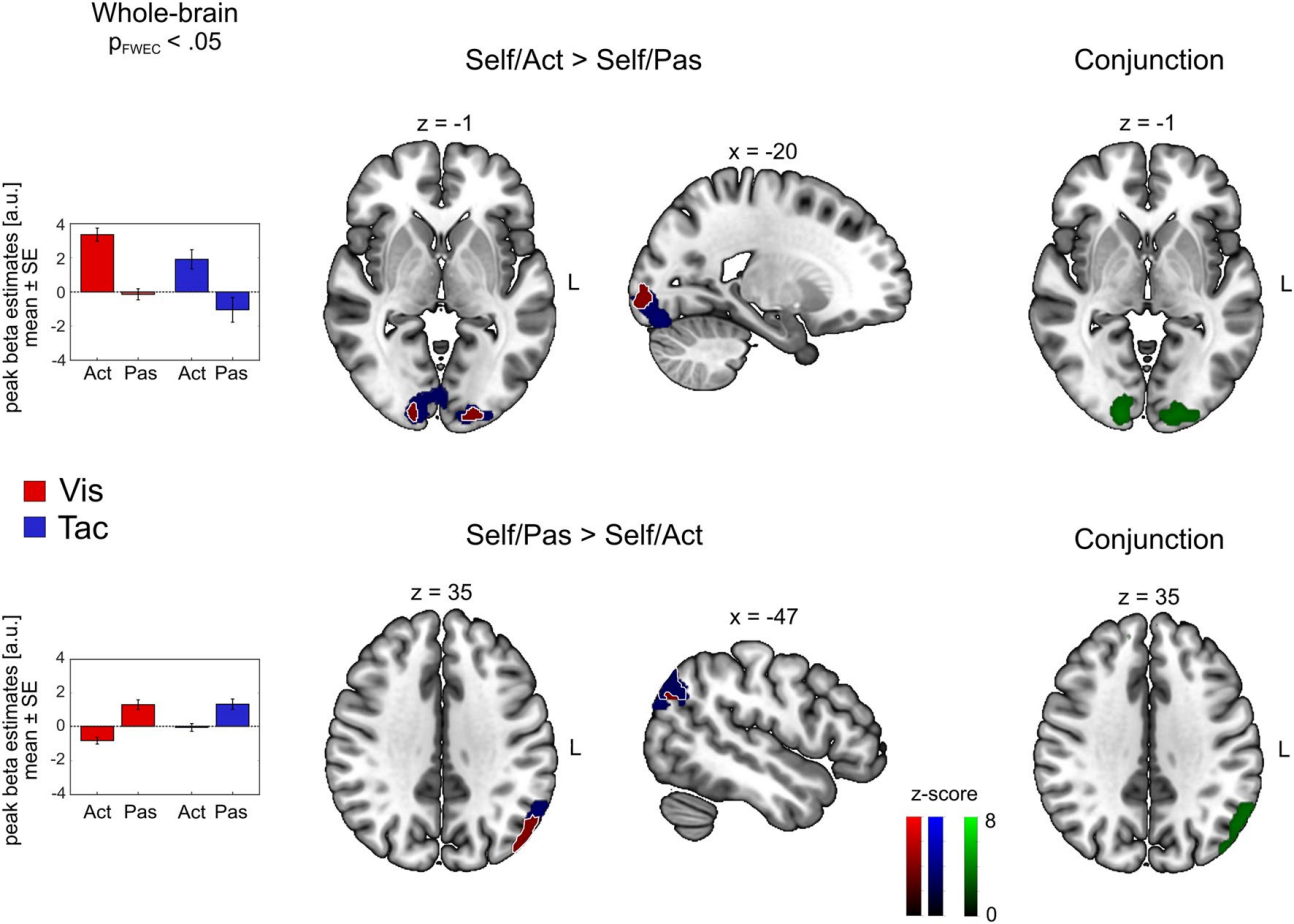
Modulatory effect of behavioral demand. Whole-brain results showing BOLD enhancement (top row) and BOLD suppression (bottom row) during the travel of self-chosen distances compared to passively observing replayed distances for visual (red) and tactile (blue) trials. Commonly enhanced or suppressed regions across visual and tactile trials are demarcated by white lines. Bar graphs show mean beta estimates across subjects (± *SE*) for the corresponding peak voxel. Clusters derived from a conjunction analysis across both modalities are shown on the right (green). Coordinates are listed in MNI space. Initial search threshold was *p* < 0.001, only regions passing the *p*_FWE_ < 0.05 at the cluster level are shown.

In the Self Task, contrasting Act against Pas trials (*Act* > *Pas*) for the visual and tactile modality yielded bilateral significant clusters in early visual and somatosensory cortices, respectively (Fig. 4, upper row). These clusters also showed significant activation in a conjunction analysis over both modalities (Self/*Act* > Self/*Pas* visual ∩ Self/*Act* > Self/*Pas* tactile). For the tactile modality we additionally found enhanced activation in the left premotor cortex and the right precuneus. Contrasting the passive against the active condition (*Pas* > *Act*) resulted in a significant cluster in the IPL for both visual and tactile trials (bottom row). A conjunction analysis across both modalities (Self/*Pas* > Self/*Act* visual ∩ Self/*Pas* > Self/*Act* tactile) identified significant activation in the angular gyrus (Fig. 4, lower row). Table 3 reports significant cluster peaks of the contrasts derived from the Self task.

**Table 3:** Anatomical locations of cluster activations for contrasts of interest in the Self task and the comparison between Repro/Act and Self/Act trials. Coordinates are listed in MNI space. Initial search threshold was *p* < 0.001, only regions passing the *p*_FWE_ < 0.05 at the cluster level are shown.

	Anatomical Label	Cluster extent (anatomy toolbox)	Coordinates (peak of sign, cluster, MNI)							
			Side	x	y	z	*k*_E_	*z* value	p_FWE_ voxel	p_FWE_ cluster
*Self/Act* > *Self/Pas*										
* Vis*	Calcarine	V1, V2	L	− 16	− 96	− 6	891	5.52	< 0.010	< 0.001
	Calcarine	V1, V2	R	16	− 94	− 2	719	5.22	< 0.010	< 0.001
* Tac*	Precuneus	SPL	R	8	− 72	52	519	4.76	= 0.057	< 0.010
	Precentral	Area 6d1	L	− 32	− 20	70	398	4.69	= 0.071	< 0.010
	Calcarine	V1, V2	R	16	− 94	− 4	891	4.64	= 0.086	< 0.001
	Calcarine	V1, V2	L	− 14	− 94	− 8	1235	4.53	= 0.123	< 0.001
* Conj*	Calcarine	V1, V2	R	16	− 94	− 4	286	4.46	= 0.086	< 0.050
	Calcarine	V1, V2	L	− 14	− 94	− 8	476	3.67	= 0.123	< 0.010
*Self/Pas* > *Self/Act*										
* Vis*	Angular	IPL	L	− 56	− 62	30	1817	5.47	= 0.001	< 0.001
	Frontal Sup II	Area p32	L	− 14	54	26	1247	4.23	= 0.202	< 0.001
	Temporal Mid	IPL	R	46	− 50	18	620	4.2	= 0.223	= 0.001
* Tac*	Angular	IPL	L	− 58	− 60	30	522	4.4	= 0.111	< 0.010
* Conj*	Angular	IPL	L	− 58	− 60	30	522	4.4	= 0.111	< 0.010
*Repro/Act* > *Self/Act*										
* Vis*	Insula	OP8	L	− 34	20	14	428	4.45	= 0.091	< 0.010
	Insula	OP8	R	32	16	10	511	4.3	= 0.160	< 0.010
* Tac*	Supp Motor Area	preSMA	L	− 6	− 2	58	376	4.43	= 0.099	< 0.001
	Insula	Area Id7	R	36	24	0	648	4.38	= 0.119	< 0.010
	Precentral L	Area 4p	L	− 32	− 20	50	340	4.34	= 0.136	< 0.050
	Frontal Inf Oper	Area 44	L	− 56	8	24	522	4.34	= 0.137	< 0.010

Activation of visual cortices in tactile trials in both the Repro and the Self task might have occurred because subjects imagined visual stimuli to solve the path-integration task in purely tactile trials. We investigated this observation further by means of a connectivity analysis (see below, section “Connectivity analysis”).

### Modulatory effect of behavioral demands

**Figure 5:**
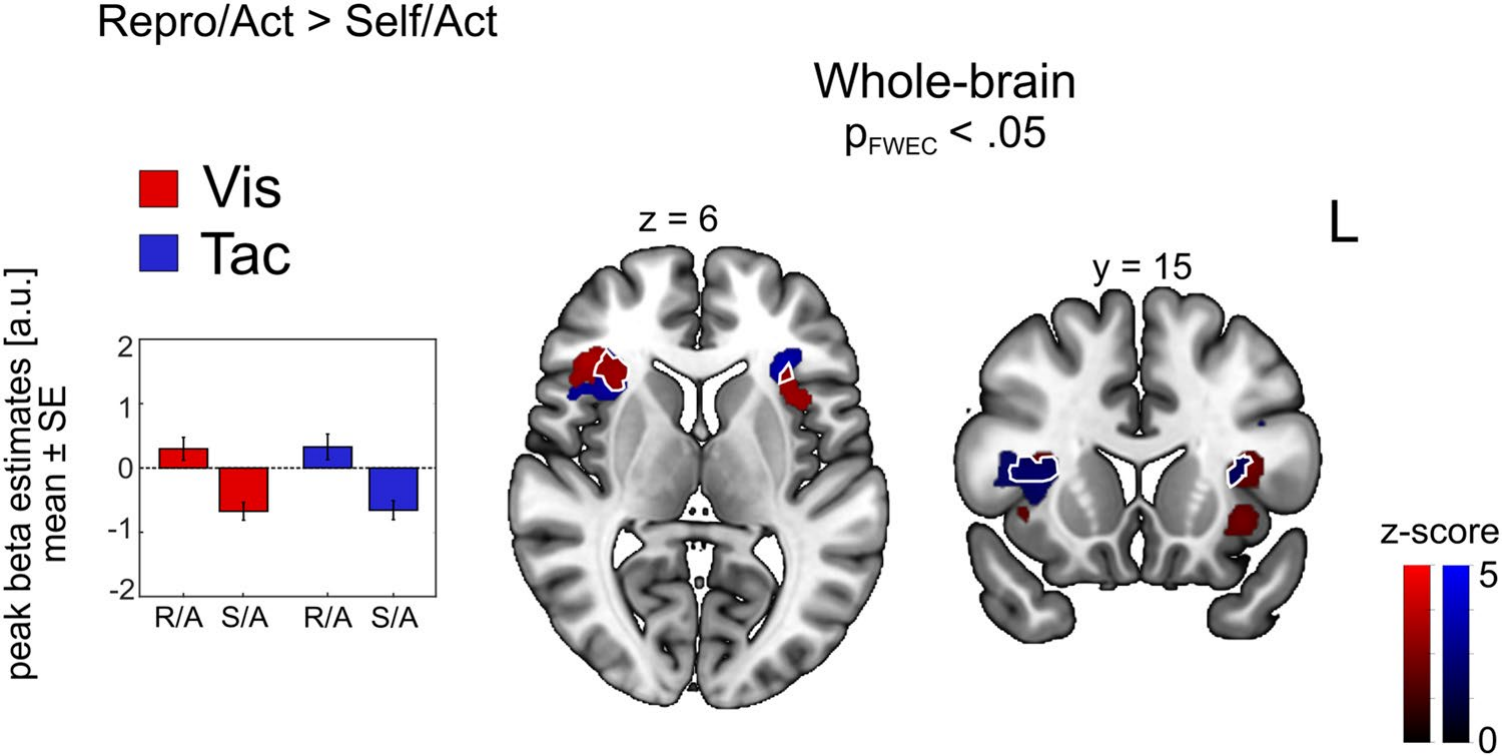
Comparison of BOLD contrast in Act trials between the Repro and the Self task. Whole-brain results show BOLD enhancement during the active reproduction compared to the production of self-chosen distances for visual (red) and tactile (blue) stimuli. Commonly enhanced regions across visual and tactile trials are demarcated by white lines. Bar graphs show mean beta estimates across subjects (± *SE*) for the corresponding peak voxel. Cluster-forming threshold was *p* < 0.001 uncorrected, with clusters significant at *p*_FWEc_ < 0.05 shown.

We investigated potential differences in BOLD response between the active reproduction of distances (Repro/Act, *high* monitoring demand) and the active production of self-chosen distances (Self/Act, low monitoring demand) by contrasting trials of both tasks (Repro/*Act* > Self/*Act*). For both modalities, we found significant clusters in the anterior insular cortex (Fig. 5). Beta estimates derived from the cluster peaks suggest enhanced BOLD response during active reproduction and suppressed BOLD response during the travel of self-chosen distances for both sensory modalities, indicating a modulatory effect of behavioral demand. Enhanced activation induced by both modalities was found in different subdivisions of the anterior insular cortex (AIC). Further significant clusters for the tactile modality were located in the motor cortex (primary motor cortex and pre-supplementary motor area). Table 3 shows the coordinates of peak clusters. The reverse contrast (Self/*Act* > Repro/*Act*) did not result in any significant clusters. There was no overlap in brain regions relevant for enhancement within the Repro conditions and across task conditions (Repro/*Act* > Repro/*Pas* ∩ Repro/*Act* > Self/*Act*).

Both tasks have also been presented in a bimodal condition where visual and tactile stimuli presented congruent distances. We also tested for multimodality effects (Bi > Sum(Vis,Tac) implemented in SPM as a weighted contrast of [2-1-1]) in all contrasts of interest. Results are shown in Supplementary Fig. S1 and Supplementary Table S1. Notably, none of the contrasts revealed significant activation differences. Thus, in our study, bimodal encoding appears to be comparable to the sum of both modalities. For the comparison between Act trials of both tasks (‘Repro/*Act* > Self/*Act*’ and vice versa) we did not find significant activation differences.

### Connectivity analysis

**Figure 6:**
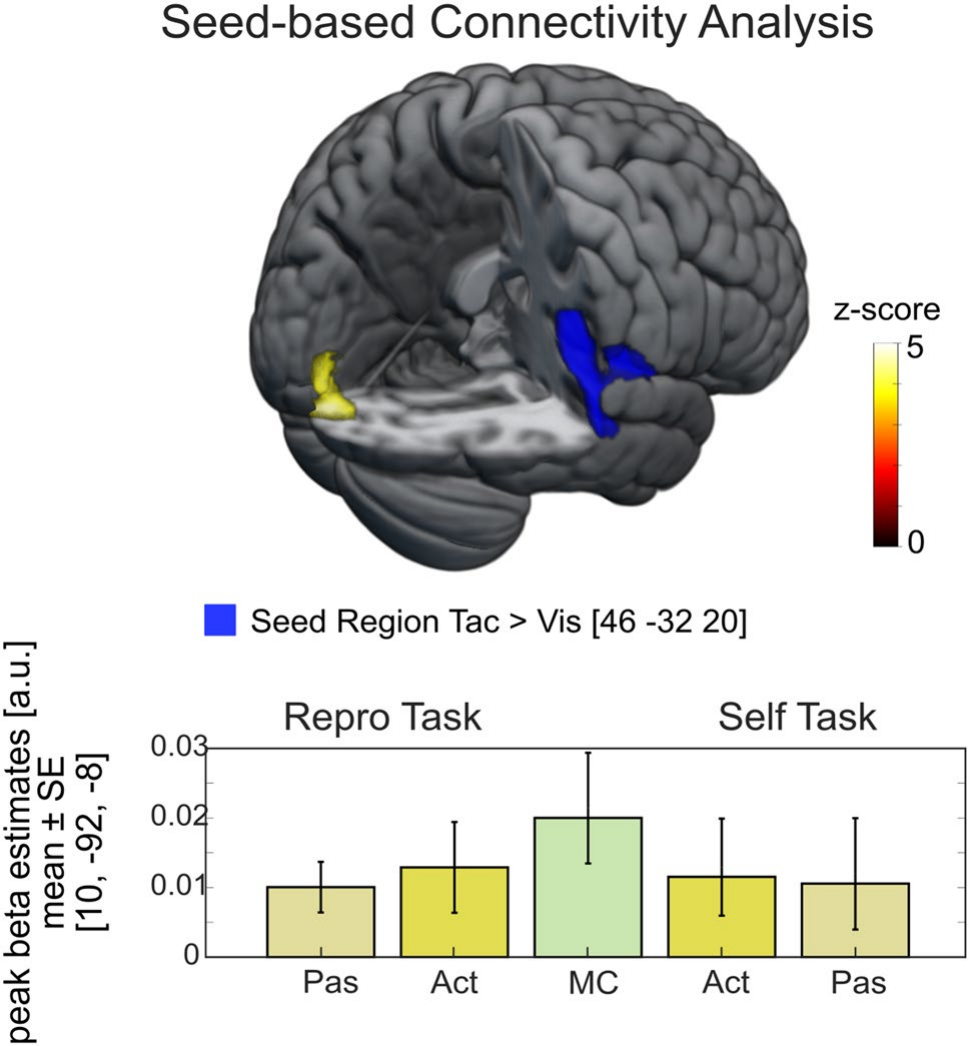
PPI whole-brain analysis depicting significant connectivity between the (tactile) seed region and visual areas as identified by a F-test. The tactile seed region in the right Parietal Operculum (MNI = − 50, − 34, 16), derived from enhanced activation in all tactile compared to all visual trials irrespective of the task, showed significant connectivity with early sensory areas (Occipital Pole; MNI = 10, − 92, − 8) for both, Act and Pas trials in both tasks (Repro, Self). Threshold: *p*_*FWE*_ < 0.05. Bar graphs show mean beta estimates across subjects (± *SE*) for the corresponding peak voxel.

To further investigate the unexpected finding of activations in visual cortex during tactile trials in both the Repro and the Self task, we conducted a PPI analysis (i.e., Psychophysiological Interactions) using the CONN toolbox (web.conn-toolbox.org/). We performed a whole-brain seed-based functional connectivity analysis by using a seed region derived from contrasting all tactile with all visual trials irrespective of the task [*Tac* > *Vis*; Seed region: Right Parietal Operculum; [*x, y, z*] = − 50, − 34, 16]. On the first level, eigenvariates extracted from the seed region created PPI regressors for all conditions of interest (Repro/*Act*, Repro/*Pas*, Self/*Act*, Self/*Pas* and the MC condition). Then we performed a whole-brain analysis to identify areas in which functional connectivity with our seed region was modulated by the type of task(-demand). Seed-based analysis results were subject to a family-wise error correction for multiple comparisons following the Gaussian Random field theory for the parametric test^29^. Figure 6 depicts the results of the connectivity analysis with corresponding beta values for the peak voxel for each effect of interest separately.

On the whole-brain level, a significant F-test identified that connectivity between the seed region (blue cluster) and the occipital pole (yellow cluster differed significantly between task conditions (Left: V1, V2; [*x, y, z*] = 46, − 32, 20, *Z* = 4.21, *k*_*E*_ = 212, p_FWEc_ < 0.05). To identify task condition dependent differences, we performed post-hoc t-tests on beta estimates derived from single task conditions. Positive beta estimates suggest connectivity between the seed region (tactile processing area) and visual areas during the passive encoding (Repro/*Pas*) and passive observation of traveled distances (Self/*Pas*). Compared to passive trials, the active reproduction of distances revealed higher beta estimates. However, the motor conditions revealed highest beta estimates and post-hoc t-tests showed that beta estimates between Repro/*Act* and MC trials (*t*(20) = − 0.943, *p* = 0.357) and between Self/*Act* and MC trials (*t*(20) = 0.94, *p* = 0.358) did not differ significantly.

To test for laterality, we have also entered the right frontal operculum as a seed region ([*x, y, z*] = 46, − 32, 20) into a PPI analysis. The seed region was defined based on the most significant right hemispheric peak cluster derived from the *Tac* > *Vis* contrast. The right frontal operculum showed significant connectivity to the bilateral calcarine cortex (V1, [*x, y, z* = 10, − 78, 0], *p*_*FWE*_ < 0.05).

## Discussion

We investigated the neural correlates of visual and tactile distance encoding (path integration) during (simulated) self-motion. We employed two types of path-integration tasks differing in behavioral demands during active distance re-/production. For both tasks and for both sensory modalities (visual, tactile) we found an enhancement (Act > Pas) in early visual areas and suppression (Pas > Act) in higher order areas of the inferior parietal lobule (IPL). Suppression in areas of the IPL suggests this area to be a comparator of predictions and incoming self-motion signals. Task demand (Act: Repro > Self) was related to enhanced BOLD response in the anterior insular cortex across modalities. We conclude that the effect of action on sensory processing is, first, supramodal and, second, more complex than previously assumed as it is dependent on task demand and the signal processing stage.

In the Repro task (high demand), subjects were presented with two different target distances that had to be reproduced. The accuracy of distance reproduction did not differ between the visual and the tactile modality. While the short target distance was replicated rather accurately, the longer target distance was often overestimated (i.e., subjects drove a longer distance than presented). Previous studies on the reproduction of traveled distance have shown accurate reproduction performance for self-motion stimuli from the visual (e.g. Ref.^30^ and auditory modality (e.g., Ref.^12^). Overestimation of travel distances might have occurred because our tasks required a comparison of the already traveled distance with the remaining distance until the target distance is reached^31,32^. Overall, our results show that also tactile self-motion stimuli allow for the estimation of travel distance.

In line with our previous study^13^, for the visual modality, we found BOLD enhancement in early visual cortical areas and BOLD suppression in higher-order areas located in the IPL for Active compared to Passive trials. For the successful reproduction of a previously observed target distance, it is necessary to first encode the target distance and to maintain it in the working memory to be able to recall it during reproduction. Hence, this task induces a high behavioral demand. BOLD enhancement in Repro/*Act* compared to Repro/*Pas* is in line with our hypothesis that active trials.

Surprisingly, we found a comparable pattern of BOLD responses in visual cortices for the tactile modality. Here, active reproduction yielded enhanced BOLD response not only in somatosensory cortical areas (SI and SII), but also in the early visual cortex as well as suppressed BOLD response in higher-order visual areas that largely matched the BOLD responses in the visual modality. Additionally, area V3A showed enhanced BOLD response during tactilely based active distance reproduction. Previous studies in humans^33^ and non-human primates^34^ demonstrated neural selectivity for visual motion of area V3A (However, see also Orban et al.^35^ who showed stronger motion sensitivity in human area V3A than monkey area V3A). A role for the processing of tactile motion so far has not been described. A conjunction analysis between contrasts of interest for visual and tactile stimulation emphasized the similarities of distance processing in the visual and the tactile modality by displaying V1 and V3a as joint regions of enhanced BOLD activation for Act compared to Pas trials and IPL as a common region for suppressed BOLD response. Our results might suggest a supramodal processing of self-motion cues in the V1, V3A and IPL during the Repro task. Alternatively, activation of areas V1 and V3A could also have resulted from imagery of the visual self-motion stimulus^36,37^. Previous work has shown that visual imagery can activate similar (if not identical) regions as real visual self-motion stimuli^36^. More recent work has revealed layer specific differences between visual stimulation and imagery in early visual cortex^38^. Yet, our approach did not allow for such fine grain analysis. Hence, our observed BOLD activation might be also, at least in part, related to visual imagery. Likewise, action patterns are thought to be stored in memory in the form of (movement) models (see e.g., Refs.^39,40^). Action imagery has also been found to be represented in motoric memory^41^. In that sense, neural activation in our study might be attributable to the imagination of inducing self-motion through one’s own action. Along this line, studies by Rieger and colleagues demonstrated similar activation patterns for the imagination and execution of a specific action^38,39,42^, for a review see Ref.^40^. In summary, neural activation in our study might have been due to imagery of self-motion and/or its control in cortical regions also responding to visual self-motion information. Future studies will be necessary to disentangle both phenomena.

In order to better understand the observed activation of early visual cortex by tactile stimuli, we conducted a connectivity analysis with a seed region derived from contrasting all tactile vs. all visual trials to achieve somatosensory activation clusters exclusively induced by tactile stimuli. This seed region was located in the right Parietal Operculum which has been shown to play a major role in somatosensory processing^43,44^. For tactile trials, at the whole-brain level, the Parietal Operculum showed positive connectivity with the left early visual cortex (V1, V2) across conditions. These results suggest that subjects might have used visual distance representations (reflected in visual cortex activation) to guide their responses to tactile self-motion stimuli.

In both modalities, higher-order areas located in the IPL exhibited BOLD suppression during the active reproduction of target distances. The IPL comprises the angular gyrus (AG), supramarginal gyrus (SMG), and the lateral intraparietal sulcus (lPS). The AG is thought, among others, to be involved in attention allocation towards task-relevant information^45,46^ and retrieved memories^47^. Human studies on path integration have described the AG to be involved in the encoding of heading stimuli^48^ and travel distance^49^. Importantly, the AG has also been described to play a role in the encoding and recall of specific paths which is also in line with the specific task presented in the Active trials of our experiment^50^. Since we found suppressed BOLD response of the AG across both modalities, our findings suggest a supramodal involvement of the AG in the encoding of target distances. This is also supported by previous studies describing activation of the AG by visuo-tactile stimuli [e.g., Ref.^51^]. The SMG has also been demonstrated to be engaged in the allocation of attention towards memory contents^52^. These areas may initiate attentional control towards the stimulus^23^ and maintain the target distance in visual short-term memory^53,54^.

Hippocampal and parahippocampal formation are involved in navigational tasks including path integration (see e.g., Ref.^55^). Hence, activation of the hippocampus and/or hippocampal formation would have been plausible. Yet, we did not find such activation. We can only speculate why this was the case. First, our task involved a virtual scenery with a ground plane composed of random dots with an unlimited lifetime. Trials were short and no landmarks were available. Hence, it appears unclear if e.g., place cells were established in this short-trial based experimental context. Second, our task was comparably easy, i.e., a single forward translation that had to be reproduced. Everyday navigational or homing tasks typically comprise translations and rotations, often with the task to return to the starting point, involving most also likely grid cell activation, which might have been absent here. Furthermore, active and passive tasks were presented from an egocentric perspective. Other than allocentric encoding, found in the hippocampal and parahippocampal formation, egocentric encoding is found in parietal cortex^56^. Overall, the task design (short trials, random dots, no landmarks, forward translation, egocentric perspective) might have contributed to the lack of hippocampal activation.

The predictive coding framework states that neural processing succeeds to distinguish self-generated from externally induced motion signals by attenuating the responses to self-generated information. In that sense, bottom-up and top-down signals interact synergistically to ensure consistent predictions at different processing levels. Predictions are defined as top-down signals that can facilitate perception and enable appropriate reactions by employing information from prediction error signals i.e., discrepancies between top-down predictions and actual incoming bottom-up sensory evidence [Refs.^57,58^, but also see Ref.^59^ who have shown that predictive information is also embedded in bottom-up processing).The fact that areas along the IPL showed suppressed BOLD activation in both modalities suggests a supramodal engagement of this region. This idea is also in line with previous findings indicating a common mechanism for processing of prediction errors in the auditory and visual modality^13,51,60^. Our study extends these findings by showing supramodal prediction processing in the IPL across the visual and tactile modality.

In the self task, the production of self-chosen distances introduced lower behavioral demand compared to the Repro task since participants were free to travel without the need to memorize and recall a given target distance. The only requirement was to stay within a certain distance limit that participants had been previously trained to maintain. Here we found a comparable pattern to our above-described results. Comparable to the Repro task, a conjunction analysis between visual and tactile trials revealed enhancement in V1 and suppression in IPL as joint areas in the processing of visual and tactile self-displacement. Suppressed BOLD activity in the IPL suggests sensory attenuation of self-generated stimuli, reflecting conformity of predictions as stated by the predictive coding framework (e.g., Ref.^61^). For both modalities, contrasting active trials with a higher behavioral demand (Repro task) with trials with a lower behavioral demand (Self task) yielded enhanced activation in bilateral AIC, indicating a supramodal engagement of the insula in solving behaviorally demanding tasks. Hence, our findings complement previous studies that found modulation of visuo-auditory AIC activation by task demands^62^ and stimulus salience^63^.

The insula, a key region in the encoding of interoceptive signaling^64^ and agency^65^ has also been shown to play a major role in the experience of time^66,67^. In our study, the participant's task was to replicate the traveled distance, not travel duration. We took several measures to ensure that subjects re/-produced distances and not durations by introducing the task specifically as a distance re/-production task. We introduced the scenario of bike riding where airflow emerges against the forehead as a function of travel direction and speed. However, responses of subjects after the experiment regarding their strategies indicated that most subjects transformed encoded distances into rhythms or paces. Most answers included ‘counting’ during self-motion in a broader sense. This indicates that the specific encoding and production of distances with a specific length might have been solved also by judging the passage of time by engaging structures like the insula. However, task instruction focused participants on the travel distance and similar optic flow and tactile flow stimuli have been shown to provide sufficient information for distance encoding^4,5^. Furthermore, in a similar behavioral visual-auditory distance reproduction task^12^, control experiments, which excluded solving the task by relying on temporal parameters, unequivocally showed that participants could solve the task by processing visual (and auditory) self-motion signals. Nevertheless, participants might still have relied, at least in part, on temporal information. Indeed, temporal processing is ubiquitous in everyday life and covers roughly twelve orders of magnitude. We can perceive differences in the order of microseconds when localizing sound^68^. At the same time, the circadian clock modulates visual processing^69^. Remarkably, the neural basis of the encoding of time in the range of hundreds of milliseconds to seconds is far from being understood^70^. At the subcortical level, the basal ganglia and the cerebellum have been implicated in temporal processing, while at the level of the cortex a whole network of regions is involved, including the visual cortex^71,72^. So, while we suggest that the observed effects were related to self-motion processing^12^, further studies are required that specifically aim to disentangle self-motion and temporal processing.

The insula has also been identified to play a role in vection^73^. In their study, Kleinschmidt and colleagues observed insular deactivation during perceived circular self-motion. Accordingly, in our study, when contrasting Repro/*Act* with Self/*Act* trials, lower beta estimates for Self/*Act* trials indicated lower activation while positive beta values for Repro/*Act* trials indicated higher activation. This activation in Repro/*Act* trials would be in line with our hypothesized BOLD enhancement due to high behavioral demand in Repro/*Act* compared to Self/*Act* trials. Importantly, the insula showed enhanced activation for both, visual and tactile trials, suggesting a supramodal mechanism of distance encoding. However, given the nature of our task, temporal aspects might have additionally contributed to AIC activation.

In our task, bimodal trials have been introduced to link visual and tactile self-motion perception. Yet, this link was not at the focus of this study. However, we also have analyzed the bimodal trials according to our contrasts of interest (Supplementary Fig. 1). Our previous observations in the unimodal conditions also apply to the bimodal condition. More precisely, for both the Repro and the Self task, Act relative to Pas trials showed BOLD enhancement of early visual cortices and suppression in higher order visual areas (Supplementary Table 1). In both tasks, visual areas are more strongly engaged during task solving which is evident from enhanced BOLD response in visual cortices in bimodal trials, where tactile information was also present. This suggests that subjects mainly relied on the visual information for solving both path-integration tasks. We also tested for multimodality effects in all contrasts of interest by investigating for an advantage of bimodal trials over the sum of both unimodal conditions. Yet, we did not find significant activation for any of the contrasts of interest. Thus, in our study, bimodal encoding can be best explained as sum of encoding in both sensory modalities.

To conclude, we have demonstrated that while self-motion signals resulted in enhanced BOLD responses in early sensory areas; this pattern was extended by insular activation if behavioral demand was high. In line with the predictive coding framework^74,75^, we found attenuated BOLD responses in the IPL, reflecting conformity of predictions (i.e., less prediction errors) and information about traveled distance. Notably, tactile path-integration was accompanied by activation of visual areas, possibly due to visual imagery.

## Methods

### Participants

Twenty-three healthy, right-handed (Edinburgh Handedness Inventory^76^) subjects participated in the study (10 females; mean age 28 years; range 20–57 years, all normal or corrected-to-normal vision) after providing written informed consent. Exclusion criteria included left-handedness, history of mental disorders, frequent alcohol or drug consumption or consumption on the day of the experiment. All subjects participated in one pretesting (behavioral pre-training) and one scanner session (on separate days). Participants received reimbursement (10€/h) after each session and were naive to the purpose of the study. Data from two participants were excluded from further analysis because of excessive head motion. In one subject, a single run out of four had to be excluded because of technical failure. All procedures used in this study were approved by the local ethics committee of the Faculty of Psychology at Philipps-University Marburg and conformed to the Declaration of Helsinki, except for pre-registration (World Medical Association; 2013).

### Stimuli and apparatus

Visual stimuli were programmed using MATLAB R2019a (The MathWorks, Natick, MA) and Psychtoolbox^77,78^. Stimuli were presented on a computer screen (LG 42 LM345, LG Electronics, Seoul, South Korea, refresh rate 60 Hz) using Octave (6.1). Participants viewed the screen via an angled mirror which covered a field of view of 21.7° (hor.) × 12.3° (vert.). Visual stimuli simulated straight-ahead self-motion across a ground plane of 2000 white random dots (luminance: 89 cd/m^2^, on a dark background: < 0.1 cd/m^2^) with unlimited lifetime, simulating self-motion with a speed of appr. 16.2 m/s. During self-motion, ground plane dots increased continuously in size when getting (virtually) closer to the observer (diameter ranged from 0.46° to 1.15°).

**Figure 7:**
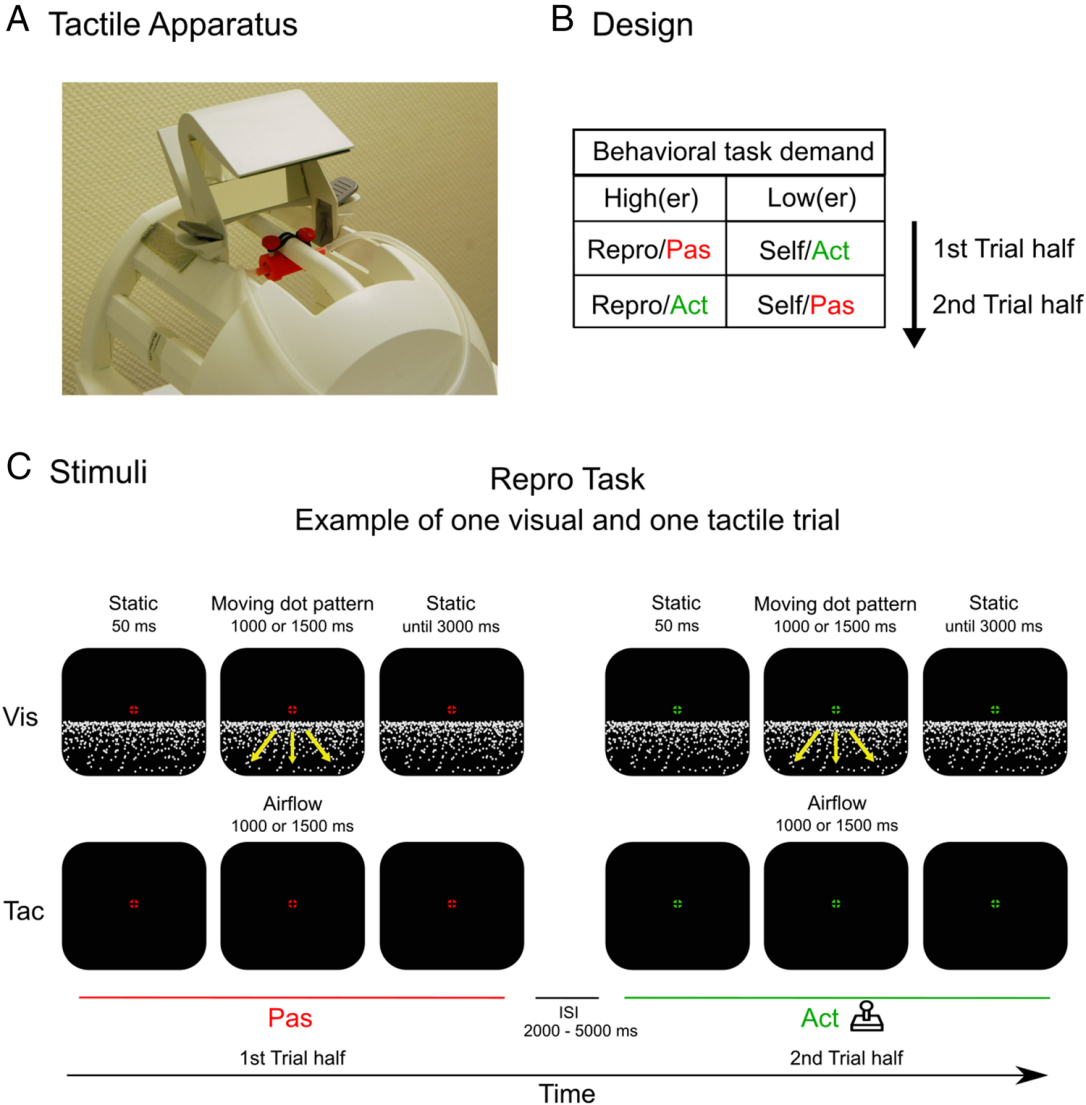
(**A**) Tactile airflow simulating forward self-motion was provided over the subjects’ forehead. A nozzle with a thin net in front of the air outlet was attached to the inner side of the head-coil and controlled by a magnetic valve. The air outlet was adjustable in tilt angle and in position on the head-coil towards the subjects’ head to ensure similar airflow position and direction for each subject. Position of the air outlet was aligned regarding subject positioning in the head coil. (**B**) Experimental design. Subjects conducted two tasks, each task in a block: The *Repro* task (higher behavioral demand) and the *Self* task (lower behavioral demand). In both tasks, a given trial always consisted of an active (Act) part and a passive (Pas) part. *Repro* task: Subjects passively observed a travel distance (Pas) which they actively reproduced (Act). *Self* task: Subjects traveled a self-chosen distance (Act) which was recorded and played back to them (Pas). (**C**) Example of a trial sequence of a Repro trial. In the Pas part, a target distance was presented that had to be replicated in the Act part by joystick deflection. A jittered ISI of 2000–5000 ms was presented between Pas and Act parts. In Vis trials, subjects were presented with an optic flow pattern simulating forward self-motion across a ground plane. In Tac trials, subjects only saw the fixation cross and felt airflow simulating a self-motion.

Tactile flow was controlled by a data acquisition system (DAQ, USB-1208FS, Measurement Computing, Sicklerville, NJ) using filtered air from a compressor (Güde Airpower 480/10/90, Wolpertshausen, Germany) which was located in a separate control room during the experiment. The DAQ was run by the Data Acquisition Toolbox for MATLAB (de.mathworks.com/products/data-acquisition.html). A nozzle attached to the inner side of the head-coil, controlled by a magnetic valve (BMT, Type AMV-MNS-24-01 (24 VDC/2W), London UK) served to provide tactile flow across the subjects’ forehead with a speed of 1.7 m/s (Fig. 7A). A thin net for air diffusion in front of the air outlet created a natural feeling of airflow. Airflow leaving the compressor was filtered and down-regulated by a pressure relief device (D-MIN-10, LUX-Tools, Wermelskirchen, Germany) before arriving at the subject's forehead (1 bar). Visual and tactile stimuli were presented with a maximum offset of 30 ms. Correct timing of airflow was constantly checked using a flow meter (Serie FCH-m-PP-LC, BIO-TECH, Vilshofen, Germany) during the whole experiment. In all conditions, participants were instructed to fixate a central target on the projection screen throughout each trial (target form specified in: Thaler et al.^79^; outer circle: 1,1° field of view, inner target: 0.28° field of view). Previous fMRI measurements with an identical visual stimulus under video-oculography^13^ and our own pilot recordings with the tactile stimulation outside the scanner and using an eye tracker established that subjects can maintain fixation over the length of time chosen for the runs (see below).

In the active condition (see below), simulated self-motion was controlled using a commercially available gamepad that was customized to the MRI environment [see Refs.^13,80^]. By forward deflection of the left analog stick of the gamepad, subjects traveled straight ahead with a constant speed. The gamepad was placed on the subjects’ upper thigh and fixed using Velcro tape. Subjects wore earplugs and MRI compatible noise-canceling headphones (Optime 1, MR confon GmbH, Magdeburg, Germany) during the whole experiment.

### Procedure

For the experiment, we introduced the scenario of bike riding and explained the analogy of airstream perceived due to air resistance of the skin against air flow. In separate trials, straight-ahead self-motion was simulated visually or tactilely. Subjects were also presented with bimodal trials where visual and tactile stimuli were presented simultaneously. However, in this manuscript we focus on unimodal distance encoding and its neural correlates only.

In both sensory conditions, each subject completed two tasks: The Reproduction task (*Repro*, higher behavioral demand) and the Self task (*Self*, lower behavioral demand). Both tasks were presented in serial order over a total of 4 consecutive runs. Task order was counterbalanced across subjects. Figure 7B illustrates the structure of trials. In both tasks, each trial consisted of an active (*Act*) part and a passive (*Pas*) part. Trials of different sensory modalities were presented in pseudorandomized order. Intertrial interval and Interstimulus interval (ISI) were randomized, ranging from 2 to 5 s.

In the *Repro* task, each trial started with the Pas part where subjects had to passively observe a traveled distance. Travel speed was always constant. To vary travel distances and to prevent subjects from learning a target distance, half of the trials involved a short distance (travel duration: 1 s), while the other half involved a long distance (travel duration: 1.5 s). Each Pas part was followed by an Act part in which the subjects replicated the previously observed travel distance by joystick deflection. They were allowed to drive a distance with a maximal duration of 3 s (twice the maximum duration in the passive displacement) until the trial ended. In the *Repro* task, we expected behavioral demand to be high given the task of continuous comparison of actively steered and passively displayed target distance. Figure 7C shows the time course of a visual (top row) and a tactile (bottom row) trial of the Repro task. After the experiment, subjects were asked to report their replication strategy, in case they had applied any.

In the *Self* task, subjects first traveled a self-chosen distance via joystick deflection in the Act part which was recorded and played back to them in the subsequent Pas part. Subjects drove at a constant speed and could travel a self-chosen distance. In a behavioral pretesting outside the scanner, subjects were trained to produce self-displacements within the range of the target distances from the Repro task. Subjects were allowed to drive a distance twice the target distance at maximum until the trial ended. When subjects overshot the target distance by more than twice the target distance, the trial ended and was counted as invalid. In the Self task, we expected behavioral demand to be lower compared to the Repro task, given that no specific predefined distance had to be reproduced.

To account for brain activation associated with the joystick deflection, we presented a motor control task (MC) after every third trial. In each MC trial, a green fixation cross was presented on the screen center with a red dot above. The red dot disappeared within a random interval between 1250 to 2250 ms. Participants were instructed to deflect the analog stick as long as the red dot was absent. After 1000 or 1500 ms, the red dot reappeared, and participants released the joystick.

Six trials per modality were presented on each run, resulting in a total of 18 trials per run plus 8 motor control trials. In total, subjects conducted 104 trials over 4 runs, with each run lasting approximately 11 min.

Before scanning, participants were invited to a behavioral training session outside the scanner to familiarize themselves with the equipment and the task. Each subject conducted two blocks (18 trials per block) of the Repro and two blocks (18 trials per block) of the Self task, each *en bloc*, plus 8 motor control trials per block.

### fMRI acquisition parameters

Functional MRI data were acquired in a Siemens 3 Tesla MR Magnetom Trio Tim scanner (Siemens, Erlangen, Germany), using a 12-channel head coil. A gradient-echo EPI sequence was used (TR: 1450 ms, TE: 25 ms, flip angle: 70° (9), slice thickness: 4 (1) mm, gap: 15%, voxel size: 3 × 3 × 4.6 mm). For each run, 350 transversal functional images were acquired in descending order. Anatomical images were obtained using a T1-weighted MPRAGE sequence (TR: 1450 ms, TE: 2.26 ms, flip angle: 9°, slice thickness: 1 mm, gap: 50%, voxel size: 1 × 1 × 1.5 mm). To minimize head motion artifacts, participants' heads were stabilized with foam pads.

### Behavioral data analysis

Analysis of behavioral data was performed using MATLAB 9.6 R2019a and SPSS (Version 23.0. Armonk, NY). For all analyses, a p-value of 0.05 or smaller determined statistical significance. For repeated measurements analyses of variance (ANOVA), Greenhouse–Geisser correction was applied to p values in case of violated sphericity assumption (Mauchly test *p* < 0.05). Effect sizes were reported by eta squared.

### Functional data analysis

Preprocessing and statistical analyses of fMRI data were performed using Statistical Parametric Mapping Version 5 (SPM12, Wellcome Department of Imaging Neuroscience, University College London, U.K.) implemented in MATLAB R2019a. The AAL atlas^81^ and the SPM Anatomy Toolbox^82^ were used for anatomical reference of significant activations. Group-level images were visualized using MRIcroGL (Version 6, www.nitrc.org/projects/mricrogl/). Effect sizes were reported as mean beta estimates using the MarsBar toolbox for SPM12 (Release 0.45, marsbar.sourceforge.net^83^). Connectivity analysis was conducted using the CONN fMRI Connectivity Toolbox (web.mit.edu/swg/software.htm), implemented in SPM12.

### Preprocessing

All scans were slice time-corrected (using the middle slice as the reference). For each run, functional images were realigned to the mean functional image of all runs. We excluded data of two participants from further analyses due to excessive head motion (translation > 3 mm). Each participant's anatomical scan was co-registered to their mean functional image and then segmented into tissue class images. The deformation field calculated in the segmentation step was used to spatially normalize the functional scans to a standard stereotaxic space based on the Montreal Neurological Institute (MNI), resampled to a voxel size of 2 mm × 2 mm × 2 mm. The volumes were then spatially smoothed using an isotropic 3D Gaussian smoothing kernel (8 mm FWHM)^84^. Functional data were analyzed using the general linear model (GLM). Low-frequency drifts were removed, employing a high-pass filter with a cutoff period of 128 s.

### First-level analysis

Regressors of interest were modeled for each run of each participant. For Act trials, contrasts were defined to account for motor-related activity by considering BOLD responses of the Motor control (MC) task: Repro/*Act* > MC and Self/*Act* > MC. In the following, MC task corrected Act trials are referred to as ‘Repro/*Act’* and ‘Self/*Act’*. In the Repro task, trials of both target distances were combined into one regressor of interest. Eight conditions of interest were defined: Repro/*Pas*, Repro/*Act*, Self/*Pas*, Self/*Act*, for each of the two modalities (Vis, Tac). Six motion parameters as well as stimulus segments that had no motion information (static dot pattern) and periods between Active and Passive trials (ITI) were modeled as regressors of no interest. Trials in which participants overshot target distances by a factor of two were excluded (1.6% of all trials).

### Second-level analysis

First-level contrasts of interest were entered into second-level random-effects analysis using a flexible factorial design and containing subjects as a random factor. Using the above-mentioned conditions of interest, we examined BOLD responses associated with distance encoding in the perception of visually and tactilely simulated self-motion, respectively, with an F-test. We assessed modulations in BOLD responses as a function of different behavioral task demand in the Repro and Self task by directional T-contrasts. For both, the Repro and the Self task, we examined BOLD enhancement effects for Act compared to Pas trials by using the T-contrasts [Repro/*Act* > Repro/*Pas*] and [Self/*Act* > Self/*Pas*]. BOLD suppression effects were assessed by the opposite contrasts [Repro/*Pas* > Repro/*Act*] and [Self/*Pas* > Self/*Act*]. All contrasts were calculated separately for the visual and the tactile modalities. To identify possible regions commonly activated during the presentation of visual and tactile modalities, we performed conjunction analyses (conjunction 0; minimum t statistic^57^).

Corresponding contrasts were also investigated for the bimodal conditions and are in the Supplementary material. Bimodal data was also investigated for multimodality effects in all contrasts of interest by testing ‘Bi > Sum(Vis,Tac)’ (Response to combined stimulation must be greater than that from a summation of the both unimodal responses) for each contrast.

To investigate possible effects of behavioral demand, differences between Act trials of the Repro and the Self task were investigated by the contrasts [Repro/*Act* > Self/*Act*] and vice versa. We expected enhanced BOLD responses in sensory cortices for the Repro- as compared to the Self task given the higher attentional and working memory demands in the Repro task.

Group-level results were visualized by reporting normalized t-values (z-scores). F-tests were calculated at the whole brain level at *p* < 0.05 family-wise error (FWE) corrected at the cluster level. For directed T-tests informed by the F-tests, we applied the following criteria: BOLD responses at the whole-brain level were assessed for statistical significance using a threshold of *p*_FWEc_ < 0.05, corrected for multiple comparisons at the cluster level with an initial search threshold of *p* < 0.001^85,86^.

## Supplementary Information

Supplementary Information.

## Data Availability

The data will be provided online at the pre-registered https://doi.org/10.17605/OSF.IO/83K92 and can be used for non-commercial research purposes upon acceptance of this article for publication.
