# Supplementary material for: Neural correlates of visual and tactile path integration and their task related modulation
Source: Sci Rep. 2023 Jun 19;13:9913. doi: 10.1038/s41598-023-36797-8 (PMC10279659; doi:10.1038/s41598-023-36797-8)
Supplement: Supplementary file 1 — Supplementary Information. [file 41598_2023_36797_MOESM1_ESM.pdf]

Supplementary Information

Neural correlates of visual and tactile path integration and their task related modulation

Lisa Rosenblum [1,2], Alexander Kreß [1,2], B. Ezgi Arikan [2,4], Benjamin Straube [2,3], Frank Bremmer [1,2]

1) Dept. Neurophysics, Philipps-Universität Marburg, Germany
2) Center for Mind, Brain and Behavior, Philipps-Universität Marburg and Justus-Liebig-Universität Giessen, Germany
3) Translational Neuroimaging Marburg, Department of Psychiatry and Psychotherapy, Philipps-Universität Marburg, Marburg, Germany
4) Department of Psychology, Justus-Liebig-Universität Giessen, Giessen, Germany

In our study, bimodal trials simulated self-motion in the visual and tactile modality. In both modalities, stimuli presented congruent travel distance. We assessed all contrasts of interest that we have reported for the unimodal visual and unimodal tactile trials for the bimodal condition as well. Fig. S1 shows significant regions for all contrasts of interest and corresponding beta values. Significant clusters are listed in Table S1.

Supplementary Table S1

	Anatomical Label	Cluster extent (Anatomy toolbox)	Side	Coordinates (peak of sign. cluster, MNI) x	y	z	*z* value	k_E
Repro/Act > Repro/Pas								
	Lingual	V1, V2, V3	L	-2	-82	16	6.48	2081
		V1, V2, V3	R	0	-80	6	6.28	409
	Cingulate							
			R	22	-46	2	5.05	22
	Lingual	V3A	R	28	-90	26	4.82	13
Repro/Pas > Repro/Act								
	Occipital Mid	hOc4lp	L	-30	-96	-6	> 8	333
			R	30	-94	-6	7.45	324
	Lateral Occipital Cortex	V5	L	-42	-66	4	6.22	120
			R	44	-62	6	5.91	676
	Supramarginal Gyrus	IPL	L	-54	-50	44	5.16	57
	Temporal Mid		L	-58	-54	2	4.83	10
Self/Act > Self/Pas								
	Lingual	V1, V2, V3	L	-18	-94	-2	6.18	293
Self/Pas > Self/Act								
	Parietal Inf	PGp	L	-44	-76	38	5.17	74

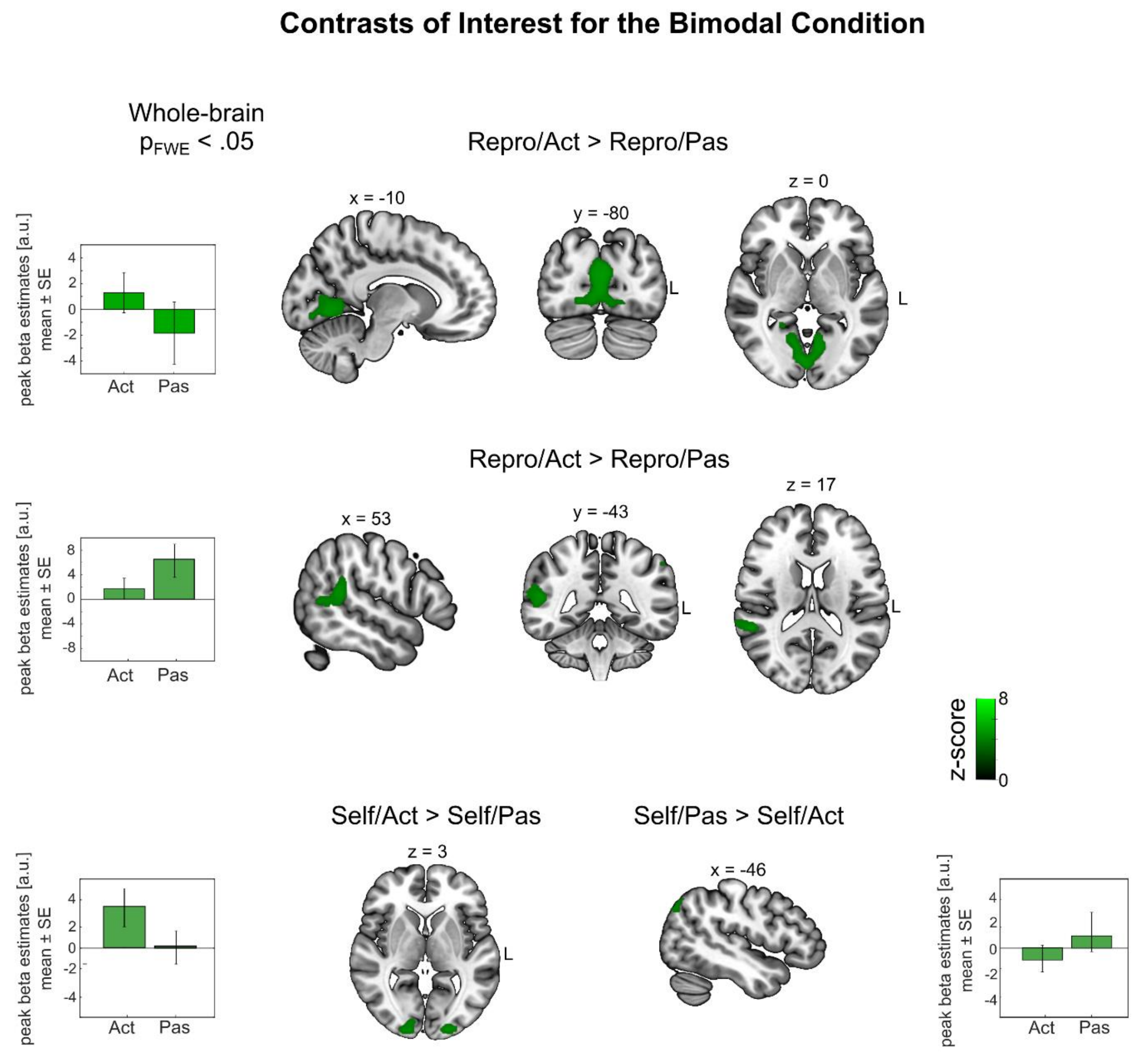

Figure S1. Modulatory effect of behavioral demand in the bimodal condition. Whole-brain results showing (i) BOLD enhancement and BOLD suppression for the Repro task (top and middle row) during the reproduction of target distances compared to passively encoding distances and (ii) for the Self task (bottom row) during the travel of self-chosen distances compared to passively observing replayed distances. Bar graphs show mean beta estimates across subjects (±*SE*) for the corresponding peak voxel. Coordinates are listed in MNI space. Cluster-forming threshold for all maps was $p_{FWE} < .05$.
